# A Whole-Food Plant-Based Diet Reversed Angina without Medications or Procedures

**DOI:** 10.1155/2015/978906

**Published:** 2015-02-10

**Authors:** Daniele Massera, Tarique Zaman, Grace E. Farren, Robert J. Ostfeld

**Affiliations:** ^1^Department of Medicine, Division of Cardiology, Montefiore Medical Center, Albert Einstein College of Medicine, Bronx, NY 10467, USA; ^2^Department of Medicine, Jacobi Medical Center, Bronx, NY 10461, USA; ^3^New York University, New York, NY 10003, USA

## Abstract

A 60-year-old man presented with typical angina and had a positive stress test. He declined both drug therapy and invasive testing. Instead, he chose to adopt a whole-food plant-based diet, which consisted primarily of vegetables, fruits, whole grains, potatoes, beans, legumes, and nuts. His symptoms improved rapidly, as well as his weight, blood pressure, and cholesterol levels. Plant-based diets have been associated with improved plasma lipids, diabetes control, coronary artery disease and with a reduction in mortality. Adoption of this form of lifestyle therapy should be among the first recommendations for patients with atherosclerosis.

## 1. Introduction

Cholesterol guidelines [1] highlight lifestyle modification as “a critical component of health promotion and atherosclerotic cardiovascular disease risk reduction.” We describe a case that reinforces this sometimes overlooked portion of the guideline's recommendations.

## 2. Case Report

A 60-year-old man presented to his primary care physician with typical angina. He reported a 1-year history of progressive severe mid-sternal chest discomfort ultimately after walking as little as one-half block, with emotional stress and in cold weather. His mother had coronary artery bypass surgery and his brother had an acute myocardial infarction, both in their early sixties.

An exercise ECG was obtained. The patient exercised for nine minutes on standard Bruce protocol. His angina was reproduced and 1.5 mm horizontal inferoapical ST depressions were noted. He declined invasive testing and he presented to our Cardiac Wellness Program at Montefiore, where he had borderline elevated blood pressure, a body mass index (BMI) of 26 kg/m^2^, elevated lipid levels, and a limited functional capacity secondary to angina (Table 1). He again declined invasive testing and despite a detailed discussion also declined drug therapy, including antiplatelet and cholesterol lowering agents.

Instead, with physician counseling, he chose to adopt a whole-food plant-based diet (WFPB), which consisted primarily of vegetables, fruits, whole grains, potatoes, beans, legumes, and nuts. He described his prior diet as a “healthy” diet of skinless chicken, fish and low-fat dairy with some vegetables, fruits, and nuts. Within a few weeks of lifestyle change his symptoms improved. After four months, his BMI fell from 26 kg/m^2^ to 22 kg/m^2^, his blood pressure normalized, and his LDL (low-density lipoprotein) cholesterol decreased from 158 mg/dL to 69 mg/dL. Previously unable to engage in physical exercise, he could now walk one mile without angina.

His clinical improvement continued and at our most recent visit, two years after initial presentation, he was able to jog more than 4 miles without incident. He remains asymptomatic, off drug therapy for coronary artery disease, and has not required cardiac catheterization.

## 3. Discussion

A whole-food plant-based diet improves plasma lipids [2], glycemic control in patients with type 2 diabetes mellitus [3, 4], reduces weight [5] and blood pressure [6–8], improves vascular function [9], may profoundly improve coronary artery disease [10–13], and is associated with reduced mortality [14–17]. Furthermore, a dose-response-like effect has been noted where the greater the adherence to a healthy lifestyle including a WFPB diet the greater the apparent benefit [18], and a growing body of evidence suggests animal based foods may not be optimal for health [19–21].

Our case reinforces these findings and highlights that even in our “modern” Western society such improvements can be achieved without medications or procedures. These results support prior epidemiologic studies which documented the virtual absence of coronary artery disease in plant-based indigenous populations, such as in parts of China [22], a highland population of New Guinea [23], the Tarahumara Indians of Mexico [24] and in South Africa [25]. Furthermore, mortality from atherosclerotic cardiovascular disease decreased when access to animal products was restricted in Norway during World War II and increased as access was returned [26]. Adoption of a plant-based diet is feasible in a real-world setting [11], not associated with markedly increased cost [27], and is successful with proper education and support [28].

## 4. Conclusion

A whole-food plant-based diet helped reverse angina without medical or invasive therapy. It appears prudent that this type of lifestyle be among the first recommendations for patients with atherosclerosis. Randomized-controlled trials are needed to further investigate this approach.

## Figures and Tables

**Table 1:** Anthropomorphic, laboratory, and clinical findings by date.

Measure	September 2012	January 2013	October 2013	September 2014
Body mass index (kg/m^2^)	26	22	21	21
Blood pressure (mmHg)	140/80	112/70	126/72	124/72
Total cholesterol (mg/dL)	234	148	125	138
Triglycerides (mg/dL)	165	155	126	120
HDLc (mg/dL)	43	34	27	36
LDLc (mg/dL)	158	83	73	78
Functional capacity	Walk 1-2 blocks	Walk 1 mile	Jog 2 miles	Jog 4+ miles

HDLc: high-density lipoprotein cholesterol; LDLc: low-density lipoprotein cholesterol.
